# Estimating the Burden of Malaria: The Need for Improved Surveillance

**DOI:** 10.1371/journal.pmed.1001144

**Published:** 2011-12-20

**Authors:** Ivo Mueller, Laurence Slutsker, Marcel Tanner

**Affiliations:** 1 Barcelona Centre for International Health Research (CRESIB), Barcelona, Spain; 2 Walter & Eliza Hall Institute, Parkville, Victoria, Australia; 3 Center for Global Health, Centers of Disease Control and Prevention, Atlanta, Georgia, United States of America; 4 Swiss Tropical & Public Health Institute, Basel, Switzerland

## Abstract

Ivo Mueller, Laurence Slutsker, and Marcel Tanner highlight the importance of using complementary methods to estimate the burden of malaria and call for a renewed focus on efficient malaria surveillance.

**Linked Research Article**

This Perspective discusses the following new study published in *PLoS Medicine*:

Cibulskis RE, Aregawi M, Williams R, Otten M, Dye C (2011) Worldwide Incidence of Malaria: Estimates, Time Trends, and a Critique of Methods. PLoS Med 8(12): e1001142. doi:10.1371/journal.pmed.1001142

Richard Cibulskis and colleagues present estimates of the worldwide incidence of malaria in 2009, together with a critique of different estimation methods, including those based on risk maps constructed from surveys of parasite prevalence, and those based on routine case reports compiled by health ministries.

Over the past decade there has been a massive scale-up of antimalarial interventions including insecticide-treated nets (ITNs), artemisinin-combination treatments (ACTs), and rapid-diagnostic tests (RDTs), and in selected areas, indoor residual spraying. This scale-up is beginning to have a significant impact on the burden of malaria in many areas worldwide. In the most recent *World Malaria Report* [1], the World Health Organization (WHO) estimated that in 2010 there was an 8% decrease in the number of cases (compared with 2005) and a 21% decrease in the number of deaths (compared with 2000). Against this backdrop of rapidly changing epidemiology, good monitoring, surveillance, and robust methods for estimating malaria burden are essential for documentation of program success as well as the identification of problem areas.

## Different Methods for Estimating Malaria Burden

In their paper published this week in *PLoS Medicine*, Richard Cibulskis and colleagues [2] compare different methods of estimating the number of malaria cases. One is the surveillance-based method used by the WHO Global Malaria Program, which estimates malaria incidence from routine surveillance reports of malaria cases compiled by health ministries (adjusted for reporting completeness, the prevalence of malaria infection among suspected cases, and the extent to which patients use public sector health facilities). The other approach uses cartographic methods as exemplified by the Malaria Atlas Project (MAP) [3],[4],[5] that combine survey data reporting malaria prevalence with case incidence from selected locations to generate global risk maps. On the whole, surveillance-based estimates of malaria burden are substantially lower than those based on risk maps, particularly in non-African regions. Both methods are subject to numerous uncertainties that affect estimates and are highly dependent on the quantity and quality of the available data.

Routine malaria case surveillance has two major advantages. Firstly, as it is collected in an ongoing manner, it allows for more real-time assessment of changes. Secondly, as surveillance is a crucial component of disease control programs, clinical and epidemiological outcomes can more easily be combined with other programmatic information to assist in planning, implementation, and modification/adjustment of malaria control activities to improve program performance. However, the accuracy of surveillance-based estimates is dependent upon the quality and coverage of the surveillance system, and in many malaria-endemic areas, current surveillance efforts are incomplete and very slow. Model-based, cartographic approaches can estimate burden in areas where (routine) surveillance is of poor quality and/or coverage e.g., where most fever cases are treated in the private sector. Because survey and case incidence studies are costly to conduct, cartographic models use data collected over longer time frames and are thus less well suited for tracking rapid, year-to-year changes in malaria burden. In addition, in areas with poor data coverage, they make inference of large geographical areas based on few, usually non-randomly selected, data points. The two methods thus have their unique strengths and weaknesses, and rather than seeing them as competing approaches, they should be synergistically combined.

## Strengthening Existing Surveillance Systems

Ultimately, good quality and up-to-date information on malaria burden will become even more important for both monitoring and operational purposes as malaria control activities are further intensified. The increased availability of RDTs will increase the proportion of parasitologically confirmed malaria cases, enhancing the quality of surveillance data, and the linkage of these case data with geographical information will allow mapping cases at the lowest possible administrative unit, facilitating the delineation of high- and low-risk areas. A further challenge will be to link surveillance efforts with the whole health system, particularly the private sector; in many countries this sector provides at least half of malaria treatments annually [6].

## Linking Surveillance to Program Outcomes

Of utmost importance, data collected by (routine) surveillance need to be “actionable”; that is, they need to be linked to program performance indicators and planning to direct program activities at district level and below, rather than simply to count and tabulate deaths, cases, or infections at different administrative levels. This will require both a review of what data should be collected and the establishment of data management and analysis capacity at all levels where operational decisions are taken. Clearly, efficient surveillance should focus on the minimal essential data required rather than the current practice that tends towards collecting the maximum amount of data possible for monitoring, evaluation, and surveillance. Surveillance systems reduced to the minimal essential data and strengthened in this way will surely improve the quality of burden estimates and, more importantly, provide essential information to enhance control and elimination activities.

## Surveillance in the Context of Elimination

As countries intensify control and further reduce transmission, programs can consider the possibility of moving towards malaria elimination. At these very low levels of transmission, surveillance activities need to be modified to become an effective tool to further reduce transmission [7]. To accomplish this, activities will need to shift from surveying deaths and cases of symptomatic clinical malaria, to detecting infections (with or without symptoms) [8]. Active and prompt detection of infection must be linked with a response package that is tailored to a given endemic setting and will help to further reduce the reservoir of infection. Response packages must include an integrated/combined manner of directed vector control and focal (or mass) screening and treatment. While much progress towards more effective surveillance can be achieved with current tools, further refinement of “surveillance as an intervention” to support malaria elimination will require improved tools and strategies such as more sensitive field-ready diagnostic tools, improved information systems, linkage of mapping with real-time surveillance data, and identification of optimal, swift, and locally appropriate, integrated response strategies.
